# Risk Factors for Intracerebral Hemorrhage: Genome-Wide Association Study and Mendelian Randomization Analyses

**DOI:** 10.1161/STROKEAHA.124.046249

**Published:** 2024-05-08

**Authors:** Susanna C. Larsson, Jie Chen, Dipender Gill, Stephen Burgess, Shuai Yuan

**Affiliations:** Medical Epidemiology, Department of Surgical Sciences, Uppsala University, Sweden (S.C.L.).; Unit of Cardiovascular and Nutritional Epidemiology, Institute of Environmental Medicine, Karolinska Institutet, Stockholm, Sweden (S.C.L., S.Y.).; Department of Big Data in Health Science, School of Public Health, Center of Clinical Big Data and Analytics of The Second Affiliated Hospital, Zhejiang University School of Medicine, Hangzhou, China (J.C.).; Department of Epidemiology and Biostatistics, School of Public Health, Imperial College London, United Kingdom (D.G.).; Department of Public Health and Primary Care (S.B.), University of Cambridge, United Kingdom.; MRC Biostatistics Unit (S.B.), University of Cambridge, United Kingdom.

**Keywords:** blood pressure, cerebral hemorrhage, genome-wide association study, risk factors, stroke

## Abstract

**BACKGROUND:**

The genetic and nongenetic causes of intracerebral hemorrhage (ICH) remain obscure. The present study aimed to uncover the genetic and modifiable risk factors for ICH.

**METHODS:**

We meta-analyzed genome-wide association study data from 3 European biobanks, involving 7605 ICH cases and 711 818 noncases, to identify the genomic loci linked to ICH. To uncover the potential causal associations of cardiometabolic and lifestyle factors with ICH, we performed Mendelian randomization analyses using genetic instruments identified in previous genome-wide association studies of the exposures and ICH data from the present genome-wide association study meta-analysis. We performed multivariable Mendelian randomization analyses to examine the independent associations of the identified risk factors with ICH and evaluate potential mediating pathways.

**RESULTS:**

We identified 1 ICH risk locus, located at the *APOE* genomic region. The lead variant in this locus was rs429358 (chr19:45411941), which was associated with an odds ratio of ICH of 1.17 (95% CI, 1.11–1.20; *P*=6.01×10^−11^) per C allele. Genetically predicted higher levels of body mass index, visceral adiposity, diastolic blood pressure, systolic blood pressure, and lifetime smoking index, as well as genetic liability to type 2 diabetes, were associated with higher odds of ICH after multiple testing corrections. Additionally, a genetic increase in waist-to-hip ratio and liability to smoking initiation were consistently associated with ICH, albeit at the nominal significance level (*P*<0.05). Multivariable Mendelian randomization analysis showed that the association between body mass index and ICH was attenuated on adjustment for type 2 diabetes and further that type 2 diabetes may be a mediator of the body mass index-ICH relationship.

**CONCLUSIONS:**

Our findings indicate that the *APOE* locus contributes to ICH genetic susceptibility in European populations. Excess adiposity, elevated blood pressure, type 2 diabetes, and smoking were identified as the chief modifiable cardiometabolic and lifestyle factors for ICH.

Intracerebral hemorrhage (ICH) is a type of stroke caused by the rupture of a blood vessel within the brain parenchyma and is related to high morbidity and mortality.^1^ Only around half of ICH patients survive the first year, and the majority of survivors are left with permanent disability.^1^ Improved insights into disease pathophysiology and the development of effective preventive and therapeutic strategies are, therefore, vastly necessitated.

ICH risk increases with advanced age, and the condition is more frequent in men, Asians, and low- and middle-income countries.^1^ Genetic susceptibility also contributes to ICH risk, but hitherto few potential risk loci have been recognized.^2–6^ A genome-wide association study (GWAS) of 1545 ICH cases and 1481 controls identified 1 susceptibility locus for nonlobar ICH (chromosomal region 12q21.1) in European ancestry individuals.^7^ Most other genetic studies on ICH have used a candidate gene approach^2–6^ and have reported a strong association with *APOE* genotypes.^5,6^ Among modifiable risk factors, hypertension and high systolic blood pressure have been strongly related to increased ICH risk in both conventional observational studies^1,8,9^ and Mendelian randomization (MR) studies based on genetic variants associated with blood pressure as instrumental variables.^10,11^ Evidence from observational and MR studies further suggests that smoking^8,9,12^ and alcohol consumption,^8,9,13–15^ 2 interrelated lifestyle behaviors, increase ICH risk. The role of other cardiometabolic and lifestyle factors, such as hyperlipidemia, adiposity, type 2 diabetes, physical activity, and sleep in causing ICH, remains unestablished.^16–26^

Available MR studies investigating the potential causal association of modifiable risk factors with risk of ICH in European populations were based on a small number of cases and ICH genotype data either from a GWAS published in 2014^7^ (comprising 1545 ICH cases and 1481 controls)^11,21–23,26^ or the UK Biobank (up to ≈1100 ICH cases).^10,13,18–20^ Here, we performed a meta-analysis of genome-wide genotype data from 3 large-scale studies and used the derived data set, comprising 7605 ICH cases and 711 818 noncases, to identify the modifiable cardiometabolic and lifestyle factors for ICH through MR analysis. We explored the independent associations for the identified risk factors and potential metabolic pathways from the risk factor to ICH through multivariable MR analysis. Although the focal point of this study was to uncover the major modifiable risk factors for ICH, the main results from the GWAS analysis are also reported.

## METHODS

### Data Availability

Summary statistics data for the GWAS meta-analysis are available at the OSF data repository (https://osf.io/s69wd/).

### Study Design

**Figure 1.**
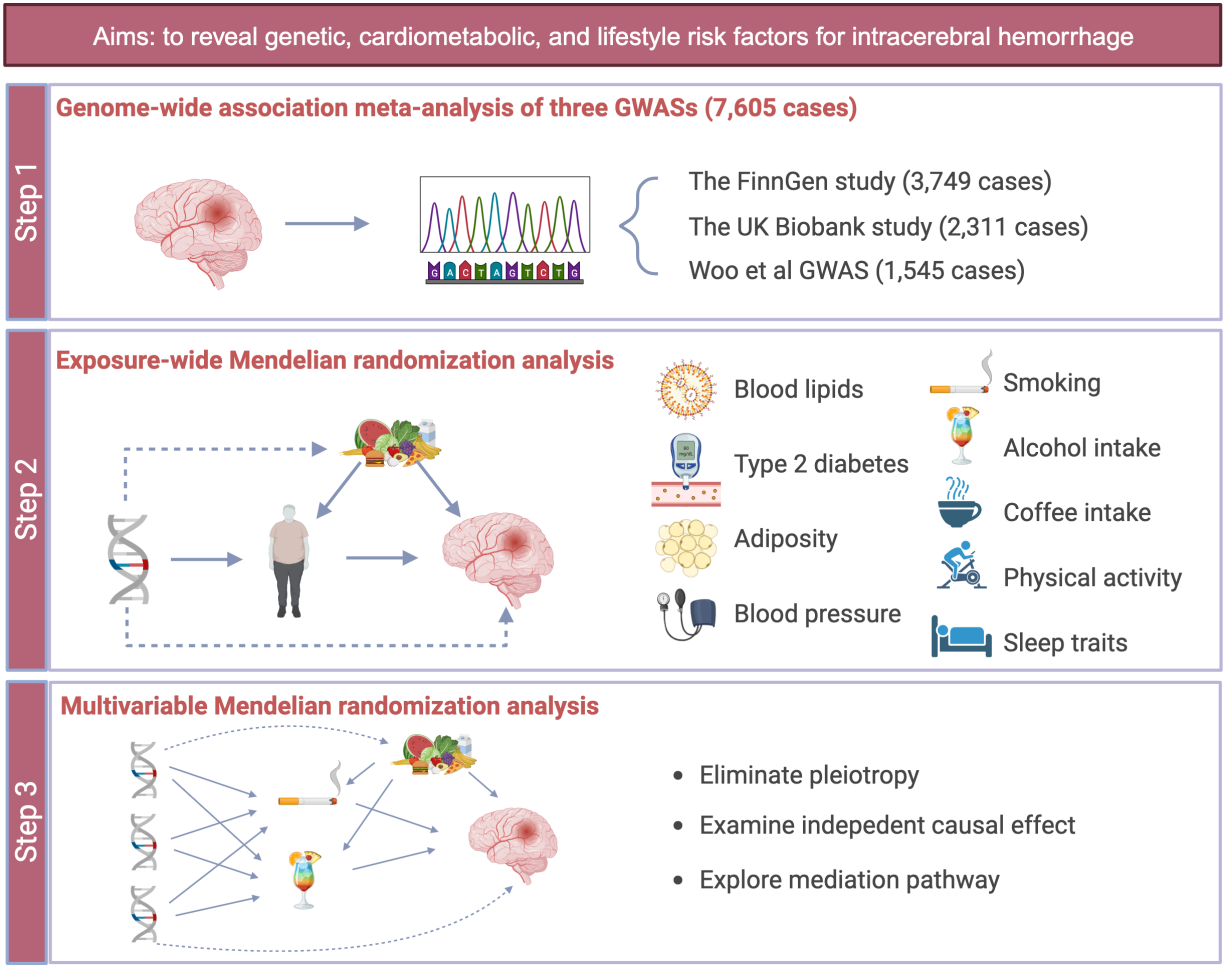
**Study design overview.** GWAS indicates genome-wide association study.

An overview of the study design is presented in Figure 1. We commenced this study by meta-analyzing GWAS data on ICH from the FinnGen study, the UK Biobank, and a previous GWAS by Woo et al^7^ in 2014. We thereafter conducted univariable MR analyses to uncover the potential causal associations of conventional cardiometabolic risk factors (ie, obesity-related traits, type 2 diabetes and major glycemic traits, blood lipid traits, and blood pressure) and major lifestyle factors (ie, smoking, alcohol and coffee consumption, physical activity and inactivity, and sleep traits) with ICH risk, using our GWAS meta-analysis data for ICH and genetic variants associated with the exposures from previous GWASs. Finally, we performed multivariable MR analyses to explore independent causal associations and mediating pathways between the identified risk factors and ICH.

### Data Sources

#### FinnGen

FinnGen is an ongoing project aimed at improving human health and promoting the discovery of therapeutic targets through genetic research.^27^ The project uses national health databases and broad genotypic data that have undergone thorough quality control measures, described in detail elsewhere.^27^ Briefly, related individuals, those with uncertain sex, non-Finnish genetic ancestry, high genotype missingness, or excess heterozygosity were removed. In the genetic variant quality control phase, variants with a missingness rate >2%, a Hardy-Weinberg equilibrium *P*<1×10^−6^, or a minor allele count <3 were excluded. Here, we utilized the ninth release of the FinnGen GWAS summary statistics data, which included 3749 ICH cases and 339 914 noncases. Cases were defined based on the *International Classification of Diseases* (*ICD*) codes: 431 for *ICD, Eighth Revision* and *ICD, Ninth Revision* and I61 for *ICD, Tenth Revision*, with data obtained from inpatient, outpatient, and social insurance reimbursement records. The association tests were adjusted for genotyping batch, age, sex, and top-10 principal components. The FinnGen study has received ethical approval, and written informed consent was obtained from all participants.

#### UK Biobank

The UK Biobank is a database and research initiative aiming to support scientific discoveries to improve human health.^28^ The cohort enrolled ≈500 000 UK adults between 2006 and 2010 and collected genetic data and information on a broad range of phenotypes. For the present GWAS, we excluded individuals lacking genetic data and those of non-British ancestry. At the sample quality control phase, we removed individuals with uncertain sex, related individuals (third-degree or closer), individuals with high missingness on genotype, and individuals with marked deviations in heterozygosity. At the variant quality control stage, we removed variants with a missingness rate >10%, variants with a Hardy-Weinberg equilibrium *P*<1×10^−6^, and variants with a minor allele frequency <0.01. ICH cases were identified from inpatient and outpatient records using *ICD* codes 431 for *ICD, Eighth Revision* and *ICD, Ninth Revision* and I61 for *ICD, Tenth Revision*. Our GWAS included 2311 ICH cases and 370 423 noncases. Associations were estimated using a logistic mixed model implemented in the REGENIE software, v2.0,^29^ and adjusted for genotyping batch, age, sex, and top-10 principal components. The study received approval from the North West Multi-Centre Research Ethics Committee. All participants provided written informed consent.

#### Woo et al GWAS

The discovery phase of the Woo et al GWAS included individuals of European ancestry from 6 studies.^7^ Spontaneous ICH cases were defined as a new and acute neurological deficit with matching brain imaging displaying the existence of intraparenchymal bleeding. The exclusion criteria included trauma, hemorrhagic transformation of ischemic stroke, vascular malformation, tumors in the brain, and other causes of secondary ICH. The control group encompassed individuals free from ICH and were recruited from the same source population as the cases. Details on quality control and imputation procedures have been described elsewhere.^7^ A total of 1545 ICH cases and 1481 controls were included in the discovery phase, which was publicly available and included in the present GWAS meta-analysis. The association tests were adjusted for age, sex, and principal components. All studies were approved by the institutional review board or ethics committee at the participating site. Informed consent was obtained from participants themselves or from their legal proxies.

### GWAS Meta-Analysis

We meta-analyzed data from the FinnGen, UK Biobank, and Woo et al GWAS under a fixed effect model and evaluated heterogeneity in the single-nucleotide polymorphism (SNP)-ICH association estimates across studies using METAL.^30^ Linkage disequilibrium (LD) score regression was applied to quantify the genomic inflation factor (λ_GC_).^31^ We used Functional Mapping and Annotation of Genome-Wide Association Studies^32^ to identify independent risk loci, performing LD-based clumping using the 1000 Genomes Phase 3 data set as the reference by setting the thresholds (the GWAS significance level at *P*<5×10^−8^, the physical distance threshold at window >500 kb, and LD thresholds at r^2^=0.6 and r_2_^2^=0.1). The lead SNP presenting the locus is defined as the variant with the lowest *P* value. The Multivariate Analysis of Genomic Annotation was used for gene mapping.^33^ For functional predictions, the tools incorporated the combined annotation–dependent depletion score, which is a metric quantifying the deleteriousness of single-nucleotide variants.^34^ Variants with elevated combined annotation–dependent depletion scores are generally considered more deleterious. We considered a locus to have a potentially deleterious functional impact when it had a combined annotation–dependent depletion score exceeding 10. To explore the pleiotropic effects associated with the identified locus/loci, we cross-referenced our findings using the GWAS Catalog database.^35^ We applied LD score regression to assess the genetic correlation between ICH and ischemic stroke and its subtypes,^36^ subarachnoid hemorrhage,^37^ and Alzheimer disease.^38^

### MR Analysis

To identify the potential causal cardiometabolic and lifestyle risk factors for ICH, we performed 2-sample MR analyses using genetic variants associated with the exposures in previous GWASs as instrumental variables and ICH outcome data from the present GWAS meta-analysis. Data sources for the exposures are detailed in Table S1. This MR investigation followed the STROBE-MR guidelines (Strengthening the Reporting of Observational Studies in Epidemiology Using Mendelian Randomization).^39^ We constructed the genetic instruments by choosing SNPs associated with each exposure at the level of genome-wide significance (*P*<5×10^−8^) in the relevant GWAS. For each exposure, we computed the LD matrix for selected SNPs using the 1000 Genomes European reference panel. For SNPs in high LD (r^2^>0.01), we kept only the SNP with the strongest association (lowest *P* value) with the exposure. We estimated the average F-statistic of used SNPs for each exposure to measure the strength of the genetic instruments. The SNPs used as instrumental variables for the exposures along with the summary statistics are provided in Table S2.

We used the multiplicative random effects inverse variance weighted method to obtain the primary MR estimates. To evaluate the robustness of these estimates, we conducted sensitivity analyses using the weighted median,^40^ MR-Egger,^41^ and MR-PRESSO (Mendelian Randomization Pleiotropy Residual Sum and Outlier)^42^ methods. We tested for heterogeneity among SNP estimates using the Cochran Q value and assessed horizontal pleiotropy using the MR-Egger intercept test and MR-PRESSO global test. To explore the independent and potential mediating effects of the exposures on ICH, we performed multivariable MR analyses. The mediation effect was calculated using the formula: (total effect−direct effect)/total effect, and the SE of the mediation estimate was calculated using the propagation of error method. Given the strong associations with ICH for several SNPs associated with the lipid traits and located near the *APOE* locus, we conducted a sensitivity analysis for major lipid traits where we excluded SNPs ±1 Mb up/down from the *APOE* locus. We corrected for multiple tests using the Benjamini-Hochberg false discovery rate approach. All MR analyses were performed with the TwoSampleMR and MendelianRandomization packages in R, version 4.1.1.

## RESULTS

### GWAS Results

**Figure 2.**
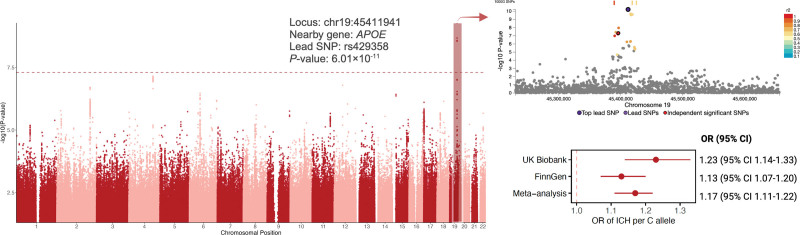
**Manhattan plot of genome-wide association meta-analysis of intracerebral hemorrhage.** ICH indicates intracerebral hemorrhage; OR, odds ratio; and SNP, single-nucleotide polymorphism.

This GWAS meta-analysis, comprising a total of 7605 ICH cases and 711 818 noncases, resulted in 1 genome-wide significant locus (*P*<5×10^−8^), located at the *APOE* locus (Figure 2). The genomic inflation factor (λ_GC_) was <1.04 in each study and 1.06 in the meta-analysis, indicating minimal inflation due to population stratification or other confounding factors. The lead variant (chr19:45411941, rs429358) associated with an odds ratio of ICH of 1.17 (95% CI, 1.11–1.20; *P*=6.01×10^−11^) per additional C allele in a meta-analysis of FinnGen and UK Biobank GWAS data (the SNP was unavailable in the GWAS by Woo et al and had no suitable proxy [LD r^2^>0.8]). The lead SNP had a combined annotation–dependent depletion of 12.6, suggesting its potential deleteriousness in ICH. A search in the GWAS Catalog database revealed pleiotropic associations of the *APOE* variant with lipid traits and Alzheimer disease, and a multitude of other phenotypes (Table S3). After correction for multiple testing, ICH showed a significant genetic correlation (r_g_) with all ischemic stroke subtypes and subarachnoid hemorrhage but not with Alzheimer disease (Table S4).

### MR Results

**Figure 3.**
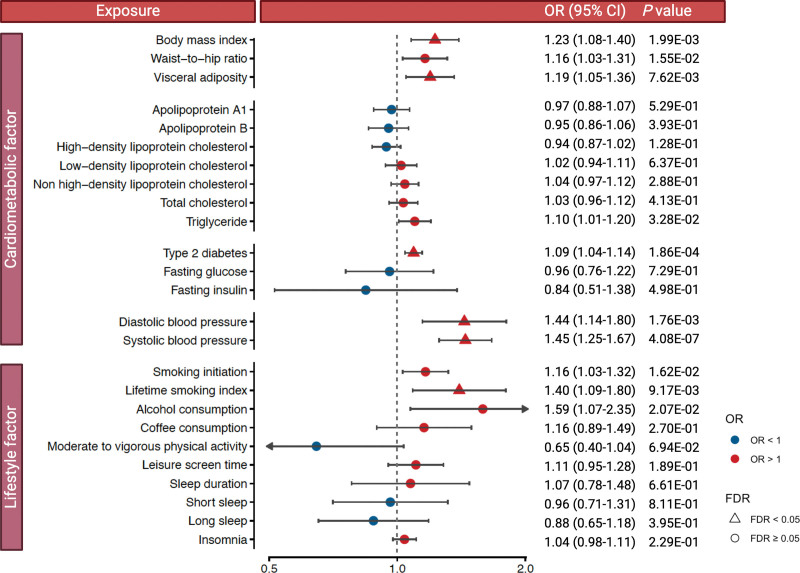
**Univariable Mendelian randomization analysis of the associations between genetically predicted cardiometabolic and lifestyle factors and intracerebral hemorrhage risk.** ORs are scaled per SD increase in genetically predicted body mass index, waist-to-hip ratio, blood lipids, and lifetime smoking index; per kilogram increase in genetically predicted visceral adiposity; per log-odds in the prevalence of type 2 diabetes and smoking initiation; per 1 mmol/L in genetically predicted fasting glucose; per 1 log-transformed picomole per liter in genetically predicted fasting insulin; per 10-mm Hg increase in genetically predicted blood pressure; per SD increase in the genetically predicted log-transformed alcoholic drinks/wk; per 50% increase in genetically predicted coffee consumption; per log-odds in moderate-to-vigorous physical activity (active vs inactive), short sleep (<7 vs 7 to 8 h/d), long sleep (≥9 vs 7 to 8 h/d), and insomnia; and per hour/day increase in sleep duration. FDR indicates false discovery rate; and OR, odds ratio.

In univariable MR analysis, genetically predicted higher levels of body mass index, visceral adiposity, diastolic blood pressure, systolic blood pressure, and lifetime smoking index, as well as genetic liability to type 2 diabetes, were associated with increased odds of ICH after correction for multiple testing (Figure 3). There was weak to moderate heterogeneity between SNP association estimates for visceral adiposity, blood pressure traits, and type 2 diabetes, but these and the associations for body mass index and lifetime smoking index remained robust in sensitivity analyses (Table S5). Genetically predicted higher levels of waist-to-hip ratio, triglycerides, alcohol consumption, and smoking initiation liability were associated with higher odds of ICH at nominal significance (*P*<0.05; Figure 3); associations for waist-to-hip ratio and smoking initiation remained consistent in sensitivity analyses (Table S5). Results for smoking initiation were consistent in a sensitivity analysis using effect size estimates (β coefficients) for the exposure from a GWAS analysis without the UK Biobank (Table S6). The association between genetically predicted alcohol consumption and ICH differed across studies, with a significant positive association in the UK Biobank, a suggestive positive association in the Woo et al GWAS, and no association in FinnGen (Table S7). We did not detect a significant association of any lipid fraction (except for the borderline association with triglycerides), fasting glucose, fasting insulin, coffee consumption, physical activity, leisure-screen time, or any sleep traits with ICH (Figure 3). The lack of significant associations for the lipid traits persisted after excluding SNPs ±1 Mb up/down from the *APOE* locus (Table S8).

**Figure 4.**
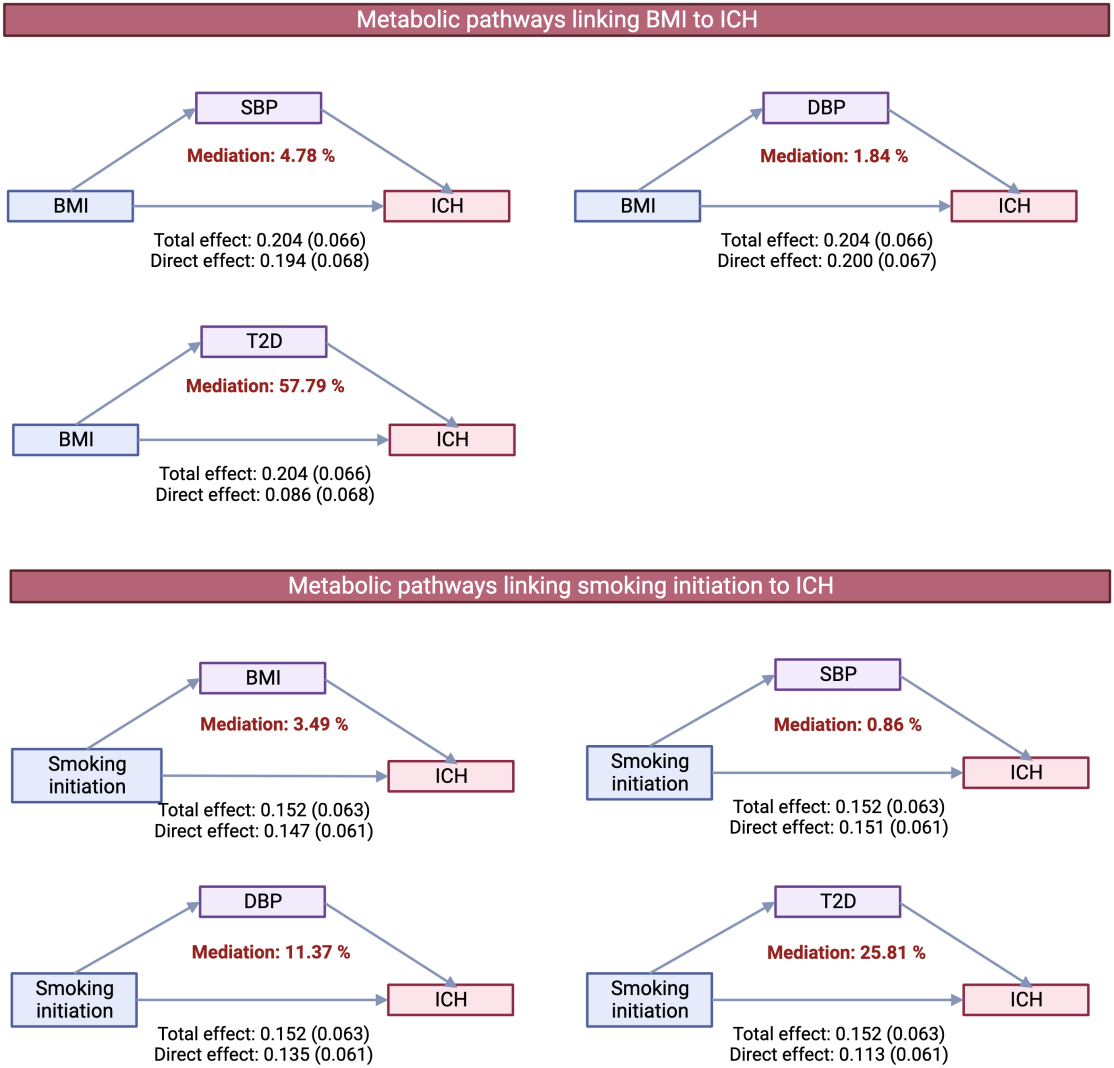
**Metabolic pathways mediating the associations of genetically predicted body mass index (BMI) and smoking initiation with intracerebral hemorrhage (ICH) risk.** DBP indicates diastolic blood pressure; SBP, systolic blood pressure; and T2D, type 2 diabetes.

Multivariable MR analysis that included various combinations of the studied exposures suggested independent associations of genetically predicted diastolic blood pressure and smoking initiation with ICH (Table S9). The multivariable MR analysis that included body mass index and type 2 diabetes revealed an attenuated association for body mass index. The mediation analysis suggested that the association between genetically predicted body mass index and ICH is to a large extent (>50%) mediated by type 2 diabetes (Figure 4) although this analysis was underpowered to detect a significant mediation effect (Table S10). There was suggestive evidence that the association between genetic liability to smoking initiation may be mediated in part by diastolic blood pressure (but not systolic blood pressure) and type 2 diabetes (Figure 4) but with broad CIs including the null (Table S9). Multivariable MR analysis of smoking initiation and alcohol consumption found that smoking was the primary trait associated with ICH in the GWAS meta-analysis of all 3 studies, but that alcohol consumption was the major trait associated with ICH in UK Biobank (Table S9). The association between alcohol consumption and ICH in UK Biobank was attenuated and appeared to be mediated to some degree by diastolic and systolic blood pressure (Table S10). The lack of association for lipid traits remained consistent in the multivariable MR analysis with mutual adjustment for the 3 lipid traits (Table S9).

## DISCUSSION

In this hitherto largest published GWAS of all ICH in individuals of European ancestry, comprising 7605 cases and 711 818 noncases, *APOE* was identified as the major ICH genetic susceptibility locus. Our MR analyses based on outcome data from this GWAS provided evidence that high blood pressure, smoking, excessive adiposity, and type 2 diabetes are risk factors for ICH.

The *APOE* gene encodes apolipoprotein E, which has a central role in lipid metabolism, both in plasma and in the brain, and is a strong genetic risk factor for Alzheimer disease.^43^ Our GWAS and previous studies based on a candidate gene approach^5,6^ indicate that the *APOE* locus also contributes to the genetic susceptibility to ICH. For example, a candidate genes study showed that the *APOE* ε2 and ε4 genotypes were associated with higher odds of lobar ICH at the genome-wide significance threshold, and the *APOE* ε2 genotype was associated with higher odds of nonlobar ICH at the level of nominal significance (*P*<0.001).^5^ A transethnic case-control study found that the *APOE* ε4 genotype was associated with higher odds of lobar ICH in white and Hispanic participants but not in black participants.^6^ The lead *APOE* variant identified in the present GWAS meta-analysis was not available in the GWAS by Woo et al,^7^ which may explain the lack of genome-wide significant association at the *APOE* locus in that study. Our GWAS meta-analysis of all ICH did not detect genome-wide significant associations with other possible risk loci (eg, *COL4A1*, *COL4A2*, 12q21.1, 1q22, *GPX1*, *CR1*, *ITGAV*, and *PRKCH*) for all ICH or its subtypes suggested by previous studies in European populations.^2,3,7,44^ As we only studied all ICH, our findings are diluted for subtype-specific loci. Given the differences in histopathology for lobar versus nonlobar (deep) ICH, further large-scale GWASs with data on ICH subtypes are needed to decipher the significance of non-*APOE* loci.

With respect to modifiable risk factors, our MR findings confirm previous data linking high blood pressure^1,8–11^ and smoking^8,9,12^ to increased risk of ICH. In our multivariable MR analysis including both diastolic and systolic blood pressure, only diastolic blood pressure remained associated with ICH. Our results provided suggestive evidence that the associations between smoking and ICH might be mediated to a minor extent by diastolic blood pressure (a nonsignificant ≈11% mediating effect) but not systolic blood pressure. Observational studies of the association between current smoking or consistent smokers and diastolic blood pressure levels have provided mixed results, with a positive,^45^ inverse,^46,47^ and no association^48^ reported. Taken together, the effect of smoking on blood pressure is at most small, and the association between smoking and ICH is likely driven by other mechanisms than by diastolic blood pressure. For example, the smoking-ICH association might, in part, be mediated by type 2 diabetes, as suggested by our MR analysis.

Self-reported high and heavy alcohol consumption, which commonly coexist with current smoking, has been associated with an increased risk of ICH in observational studies.^8,9,14,15^ In addition, genetically predicted alcohol consumption was associated with higher odds of ICH in previous MR analyses based on data from the China Kadoorie Biobank,^14^ the UK Biobank,^13^ and the Woo et al GWAS (nonsignificant association).^13^ The present MR analysis confirmed the association between genetically predicted higher alcohol consumption and ICH in the UK Biobank and the nonsignificant association in the Woo et al GWAS data set. The association between alcohol consumption and ICH in UK Biobank was independent of smoking but seemed to be partly mediated by blood pressure, as expected given the strong association between alcohol consumption on blood pressure.^13,49^ The lack of consistent association between genetically predicted alcohol consumption and ICH based on our GWAS meta-analysis data set was driven by the null association in the FinnGen study. This lack of association may suggest that the genetic instrument used for alcohol is not a good predictor of alcohol-related harm in FinnGen participants.

Obesity and type 2 diabetes are interrelated metabolic traits associated with many chronic diseases, but previous results of these traits in relation to ICH are conflicting. The INTERSTROKE case-control study that involved 663 ICH cases and 3000 controls from 22 countries found that a high (versus low) waist-to-hip ratio was associated with an odds ratio of 1.41 (95% CI, 1.02–1.93).^8^ Consistently, an observational analysis of data from the UK Biobank, including 1391 incident ICH cases ascertained among 490 071 participants, showed that waist circumference adjusted for body mass index was positively associated with ICH risk.^50^ Additionally, a positive association of genetically predicted body mass index^25^ or waist-to-hip ratio^22^ with ICH was reported in previous MR studies. In contrast, a borderline inverse association between body mass index and ICH was observed in the Million Women Study of 712 433 women (n=2032 ICH cases).^9^ Nevertheless, this study found that diabetes that required treatment was associated with a 31% higher risk of ICH.^9^ Our MR analyses found that all studied adiposity measures and type 2 diabetes liability were associated with higher odds of ICH, and the association for body mass index may be largely mediated by type 2 diabetes.

Findings for blood lipids in relation to ICH are equivocal, and if anything, the direction of associations is opposite to those for atherosclerotic diseases, such as ischemic stroke.^8,51^ Results of the INTERSTROKE study (n=469 ICH cases; n=2127 controls) showed that high levels of HDL (high-density lipoprotein) cholesterol were associated with higher odds of ICH, whereas high levels of non-HDL cholesterol were associated with lower odds of ICH.^8^ In a nested case-control study within the China Kadoorie Biobank (n=4776 ICH cases), no association was observed for HDL cholesterol, but a 1-mmol/L lowering in LDL (low-density lipoprotein) cholesterol was associated with a significant 16% higher odds of ICH in the observational analysis and a nonsignificant 13% higher odds of ICH in the MR analysis.^52^ Consistent with this, an MR study based on some studies included in the Woo et al GWAS (n=1286 ICH cases of European ancestry) reported a significant association between genetically predicted higher LDL cholesterol and lower odds of ICH.^20^ In contrast, other MR studies based on ICH data from the Woo. et al GWAS (n=1545 cases) did not detect any association for non-HDL cholesterol or triglycerides,^26,53^ but one of these studies found that higher HDL cholesterol levels were associated with significantly higher odds of ICH in a multivariable MR analysis adjusted for non-HDL cholesterol and triglycerides.^53^ An MR study that included 1064 ICH cases ascertained in the UK Biobank (n=367 703 participants) found that genetically predicted higher levels of HDL cholesterol and triglycerides were significantly associated with lower odds of ICH.^19^ In our MR analysis of blood lipids and risk of ICH, contamination of ICH with secondary hemorrhage may have biased the results towards the null.

Physical activity was not associated with ICH risk in previous observational studies, such as the INTERSTROKE study^8^ and a British cohort study,^9^ consistent with our MR finding. Studies on sleep traits in relation to the risk of ICH are scarce. We previously found that short sleep duration (<7 versus 7–9 hours/day) was associated with a 21% higher risk of incident ICH in a Swedish cohort.^21^ The present MR result for short sleep was null, but the precision of the estimate was low, with a 95% CI ranging from 0.71 to 1.31. We also found no association between genetically predicted long sleep or insomnia with ICH. As in previous observational^54,55^ and MR studies,^56^ we found no evidence of an association between coffee consumption and ICH. Hence, physical activity, sleep, and coffee consumption are not likely to be important risk factors for ICH.

Strengths of this study include the large sample size and the comprehensive exploration of the causal role of potential cardiometabolic and lifestyle risk factors for ICH. A shortcoming is that we were unable to study ICH subtypes. We, therefore, may have overlooked ICH risk loci and modifiable risk factors related to only lobar or nonlobar ICH. Another limitation is the *ICD*-based definition of ICH and the lack of case adjudication in FinnGen and UK Biobank. This likely increased study heterogeneity through the inclusion of at least some degree of miscoded cases with secondary hemorrhage due to head trauma, brain tumor, subarachnoid hemorrhage, and hemorrhagic transformation of ischemic strokes. Another weakness is that we were unable to explore sex-specific associations due to a lack of sex-specific data on ICH. Furthermore, as we confined our study sample to individuals of European ancestry (to diminish population stratification bias), our results may not be transferable to non-European populations. Finally, we had no replication sample to validate the identified risk locus. However, the genomic region identified in the present GWAS has been reported in previous candidate genes studies,^3^ and our study may, thus, be viewed as a replication of prior findings.

### Interpretations

Our study corroborates the reported association between *APOE* and the risk of ICH in populations of European ancestry. Maintaining a healthy blood pressure throughout life and avoidance of excessive adiposity, type 2 diabetes, and smoking were prioritized as the chief modifiable risk factors for ICH.

## ARTICLE INFORMATION

### Acknowledgments

The authors acknowledge participants and investigators of the FinnGen and UK Biobank studies and investigators of the Woo et al GWAS for sharing summary-level data. The analysis of UK Biobank data was conducted under application number 29202. Drs Larsson and Yuan conceived and designed the study and created the data visualizations. Drs Chen and Yuan undertook the statistical analyses. Dr Larsson wrote the first draft of the article. All the authors contributed to data interpretation, offered significant intellectual insights to the article, and approved the final version.

### Sources of Funding

This work was supported by the Swedish Research Council for Health, Working Life and Welfare (Forte; grant 2018-00123); the Swedish Research Council (Vetenskapsrådet; grant 2019-00977); and the Swedish Heart-Lung Foundation (Hjärt-Lungfonden; grant 20190247). Dr Burgess was supported by the Wellcome Trust (grant 225790/Z/22/Z) and the UK Research and Innovation Medical Research Council (grant MC_UU_00002/7). Dr Yuan was supported by the American Heart Association Postdoctoral Fellowship (grant 24POST1189614).

### Disclosures

None.

### Supplemental Material

STROBE-MR Checklist

Tables S1–S10

## Supplementary Material


